# Effects of Obesity on Bone Healing in Rats

**DOI:** 10.3390/ijms222413339

**Published:** 2021-12-11

**Authors:** Anna Damanaki, Svenja Memmert, Marjan Nokhbehsaim, Ali Abedi, Birgit Rath-Deschner, Andressa Nogueira, James Deschner

**Affiliations:** 1 Department of Periodontology and Operative Dentistry, University Medical Center, University of Mainz, 55131 Mainz, Germany; aa_abedi@yahoo.com (A.A.); a.nogueira@uni-mainz.de (A.N.); james.deschner@uni-mainz.de (J.D.); 2 Center of Dento-Maxillo-Facial Medicine, Department of Orthodontics, University of Bonn, 53111 Bonn, Germany; svenja.memmert@ukb.uni-bonn.de (S.M.); brathde@uni-bonn.de (B.R.-D.); 3 Section of Experimental Dento-Maxillo-Facial Medicine, Center of Dento-Maxillo-Facial Medicine, University of Bonn, 53111 Bonn, Germany; m.saim@uni-bonn.de

**Keywords:** periodontal regeneration, obesity, fenestration, animal, periodontitis

## Abstract

Although the association between periodontitis and obesity is well explored, it is unclear whether obesity is associated with a worse therapeutic outcome after periodontal treatment. The aim of this study was to investigate the effects of obesity on bone healing with and without the application of regeneration-promoting molecules. A standardized bone fenestration-type defect was created over the root of the mandibular first molar in 15 Wistar rats. Ten animals received a high-fat, high-sucrose diet (HFSD), while the remaining five animals were fed a standard diet. During surgery, the fenestration defects from half of the HFSD-fed, i.e., obese animals, were treated with regeneration-promoting molecules (enamel matrix derivative; EMD). After four weeks, bone healing was evaluated by histomorphometry, TRAP staining and immunohistochemistry for RUNX2 and osteopontin. The analyses revealed that the spontaneous healing of the periodontal defects was compromised by obesity. Application of EMD partially compensated for the negative effect of obesity. Nevertheless, EMD-stimulated bone healing in obese animals was not better than the spontaneous healing in the obesity-free control group, indicating that obesity may also inhibit the stimulatory effects of regeneration-promoting molecules. Our results show that obesity can negatively influence bone healing and suggest that bone healing may be compromised in humans.

## 1. Introduction

Periodontitis is a chronic multifactorial inflammatory disease that affects the periodontium, i.e., the tooth-supporting tissues, and can lead to tooth loss if left untreated [1,2,3]. Bacteria in a dysbiotic subgingival biofilm induce an immunoinflammatory response, but this host reaction is too long, strong, weak or misdirected. As a result, the bacteria, their constituents and products, and the immunoinflammatory processes further degrade subgingival structures of the periodontium, including the periodontal ligament and alveolar bone, clinically characterized by periodontal pockets, bleeding on probing, increased tooth mobility and migration and tooth loss [1,2,3]. The prevalence of periodontitis worldwide is high, placing this disease in the sixth position of the most prevalent conditions affecting humans, and rising due to the increase in life expectancy in many countries [4]. Strong evidence demonstrates that periodontitis not only affects masticatory function, aesthetics and self-esteem, but that chronic periodontal inflammation also exerts a negative effect on numerous diseases of the whole organism [5,6]. Thus, it is known that periodontitis is causally associated with increased intima-media thickness of the carotid artery, poorer endothelial function of the brachial artery, increased pulse wave velocity, cardiovascular diseases, obesity, hyperlipidemia, diabetes mellitus, rheumatoid arthritis, as well as neurodegenerative and other diseases [7,8,9,10,11,12,13].

The periodontium consists of gingiva, periodontal ligament (PDL), root cementum and proper alveolar bone. In periodontitis, destruction or alteration of all these periodontal components occurs. The primary goal of periodontal therapy is to reduce the periodontal pathogenic bacteria to the level where the host can successfully control the remaining bacteria again, thereby stopping or at least delaying the progression of periodontitis. This is usually achieved successfully by subgingival instrumentation of the root surface using hand, sonic and/or ultrasonic instruments, alone or in combination with adjuvant systemic antibiotic administration [14]. The outcome of such conservative non-surgical periodontal therapy is usually characterized by repair of the periodontal structures [15]. However, an optimal therapeutic outcome would be periodontal regeneration, i.e., complete restoration of the original function, shape and structure of all periodontal tissues. This can usually only be achieved with specific regeneration-promoting molecules and/or structures for certain defect morphologies [16].

Enamel matrix proteins belong to the molecules that can promote periodontal regeneration [17]. These can be extracted from porcine tooth germs and commercially purchased as enamel matrix derivative (EMD) for clinical use [18]. Several preclinical and clinical studies have demonstrated that EMD promotes periodontal regeneration [17,19,20,21,22,23,24,25,26].

Obesity is characterized by excessive fat accumulation with pathological effects, increasing prevalence worldwide, and adverse effects on numerous diseases, e.g., cardiovascular diseases, type II diabetes, and cancer [27]. Numerous mechanisms have been described for the negative impact on the whole organism, with elevated levels of lipids and adipokines playing an important role [28]. Since periodontitis is associated with cardiovascular disease and diabetes mellitus, it has also been investigated whether periodontitis is directly related to obesity [8,10]. The meta-analyses clearly demonstrate that obese individuals are more likely to develop periodontitis and that periodontitis patients are on average more overweight or obese [29,30]. Although further research is needed to clarify the causality of this association, numerous pathomechanisms have been described that could very plausibly explain how obesity might contribute to an increased risk of periodontitis and vice versa [31]. In addition, although causality is very likely, there are also common risk factors that could contribute to the association between periodontitis and obesity, such as age, socioeconomic position and alcohol [31].

Thus, since obesity can be considered a risk factor for periodontitis, the question was raised whether obesity could also affect the response to periodontal therapy. Surprisingly, meta-analyses have not yet been able to clearly answer this question, although one would actually assume a poorer response to periodontal treatment in obesity [32,33]. Animal studies, in which the conditions are very controlled, and the animals hardly differ, could therefore help to answer this clinically very relevant question.

Therefore, the aim of our study was to investigate the effects of obesity on spontaneous and regenerative bone healing with EMD in a controlled animal model. Our hypothesis was that obesity leads to compromised bone healing.

## 2. Results

### 2.1. Impact of Obesity on Alveolar Bone Healing in the Presence and Absence of EMD

**Figure 1:**
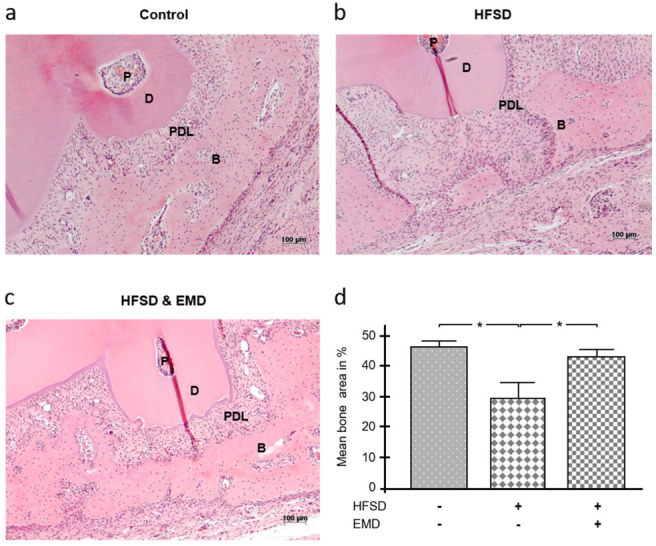
Effect of obesity on alveolar bone healing in the presence and absence of EMD. Bone healing 4 weeks after surgical creation of a fenestration defect over the root surface of a mandibular molar in normal-weight control animals (**a**), HFSD-fed animals (**b**), and HFSD-fed and EMD-treated animals (**c**). Representative images of H&E stained tissue sections from each group are shown. (**d**) The mean percentage bone area after 4 weeks of healing in control animals and HFSD-fed animals either with or without EMD treatment. Bars show mean ± SEM; *n* = 5 animals/group; * significant (*p* < 0.05) difference between groups. EMD (enamel matrix derivative), HFSD (high fat, high sucrose diet), P (pulp), D (dentin), PDL (periodontal ligament), B (bone).

After fenestration defects were created in all three experimental groups, it was assessed 4 weeks later whether spontaneous osseous healing of the defect was affected by a high-fat, high-sucrose diet (HFSD), i.e., obesity. Another objective was to evaluate whether the application of EMD, i.e., regeneration-promoting molecules, could modulate a possible effect of HFSD on bone healing. Animals fed the HFSD diet, whether treated with EMD or not, weighed significantly more than those fed a standard diet at both surgery (HFSD and EMD vs. control: *p* = 0.016; HFSD vs. control: *p* = 0.008) and sacrifice (HFSD and EMD vs. control: *p* = 0.016; HFSD vs. control: *p* = 0.016). As shown in Figure 1a, there was almost complete spontaneous regeneration of the fenestration defect over the 4 weeks following surgery. In contrast, in the HFSD group without EMD, the alveolar bone over the root surface had not yet completely regenerated but was interrupted by soft tissues (Figure 1b). In contrast, in the HFSD group with EMD application, the alveolar bone had reformed significantly better, suggesting that EMD could partially compensate for the inhibitory influence of obesity (Figure 1c). The difference between the control and HFSD groups in terms of alveolar bone was statistically significant (*p* < 0.05) (Figure 1d). Similarly, the difference between the two HFSD groups was significantly different (*p* < 0.05), i.e., the HFSD group with EMD application showed significantly more bone than the HFSD group without EMD (Figure 1d).

### 2.2. Effect of Obesity on Number of Osteoclasts in the Presence and Absence of EMD

**Figure 2:**
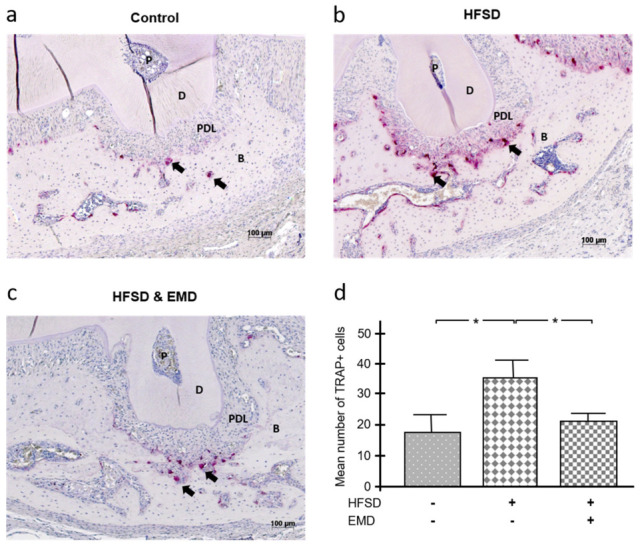
Impact of obesity on number of osteoclasts in the presence and absence of EMD. TRAP+ cells in normal-weight control animals (**a**), HFSD-fed animals (**b**), and HFSD-fed and EMD-treated animals (**c**). Shown images are representative of tissue sections stained for TRAP from all groups. TRAP+ cells/osteoclasts are indicated by black arrows. (**d**) The mean number of TRAP+ cells after 4 weeks of healing in control animals and HFSD-fed animals either with or without EMD treatment. Bars show mean ± SEM; *n* = 5 animals/group; * significant (*p* < 0.05) difference between groups. TRAP (tartrate-resistant acid phosphatase), EMD (enamel matrix derivative), HFSD (high fat, high sucrose diet), P (pulp), D (dentin), PDL (periodontal ligament), B (bone).

Tartrate-resistant acid phosphate (TRAP) is a glycosylated monomeric metalloprotein enzyme that is closely associated with migration, differentiation, activation and proliferation of osteoclasts and, therefore, with resorption or remodeling of bone. As shown in Figure 2a, only a few TRAP+ cells could be detected in the defect area on the sections of the control group. In contrast, the number of TRAP+ cells was highly increased in the HFSD group without EMD (Figure 2b). In contrast, the HFSD group with EMD showed significantly fewer TRAP+ cells (Figure 2c). The statistical analysis confirmed this observation, i.e., the HFSD group without EMD application was characterized by statistically more (*p* < 0.05) TRAP+ cells than the control group and the HFSD group with EMD treatment, as shown in Figure 2d.

### 2.3. Influence of Obesity on RUNX2 and Osteopontin Levels in the Presence and Absence of EMD

**Figure 3:**
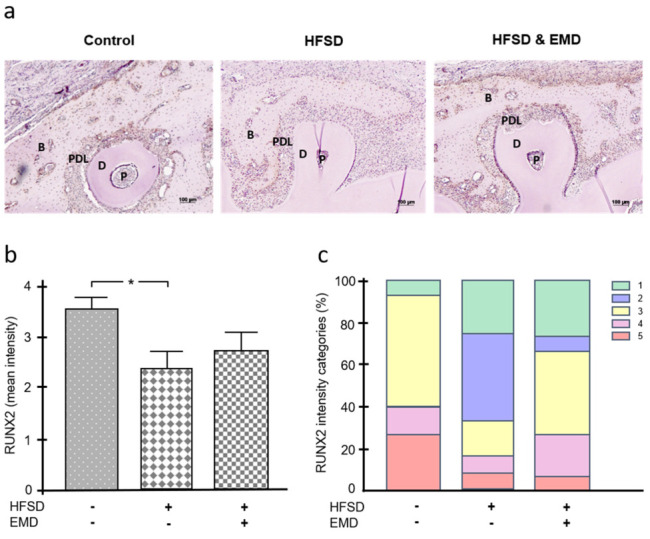
Influence of obesity on RUNX2 in the presence and absence of EMD. (**a**) RUNX2 protein in normal-weight control animals, HFSD-fed animals, and HFSD-fed and EMD-treated animals. Representative immunohistochemistry images are shown. (**b**) Mean intensity of RUNX2 in normal-weight control animals, HFSD-fed animals, and HFSD-fed and EMD-treated animals. Bars show mean ± SEM; *n* = 5 animals/group; * significant (*p* < 0.05) difference between groups. (**c**) Frequency distribution of different intensity categories for RUNX2 in normal-weight control animals, HFSD-fed animals, and HFSD-fed and EMD-treated animals. The intensity of immunohistochemical staining was assigned to five intensity categories (1 = very low, 2 = low, 3 = moderate, 4 = high, 5 = very high). EMD (enamel matrix derivative), HFSD (high fat, high sucrose diet), P (pulp), D (dentin), PDL (periodontal ligament), B (bone).

The histological sections were also incubated with antibodies against two significant molecules of bone formation and mineralization, i.e., runt-related transcription factor 2 (RUNX2) and osteopontin. The intensity of immunohistochemical staining was then classified into five intensity categories (very low, low, moderate, high, very high) and subsequently evaluated. Figure 3a shows a representative section for each group incubated with an anti-RUNX2 antibody. Figure 3b shows that the mean intensity for RUNX2 was strongest in the control group and weaker in the two HFSD groups. The difference between the HFSD group without EMD treatment and the control group was significant (*p* < 0.05) (Figure 3b). Figure 3c reveals that the higher intensity categories were numerically more frequent in the control group, which was expected due to the highest mean intensity for this group. In contrast, the lowest two intensity categories occurred most frequently in the HFSD group without EMD application (Figure 3c). The three highest intensity categories were detected twice as often in the HFSD group with EMD application as in the HFSD group without EMD.

**Figure 4:**
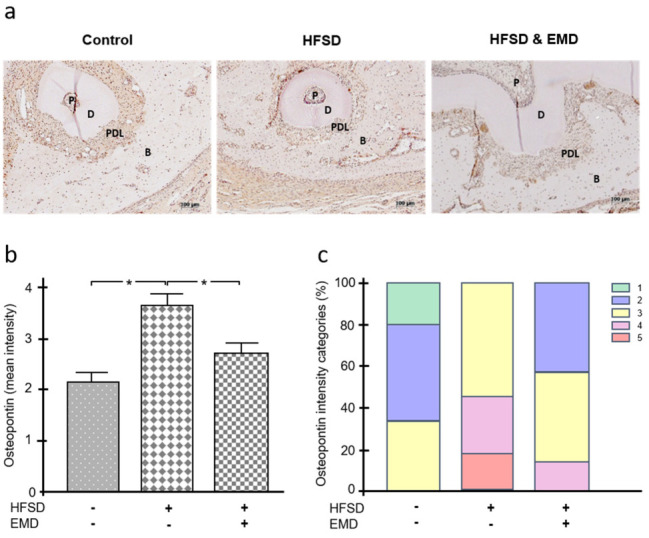
Effect of obesity on osteopontin in the presence and absence of EMD. (**a**) Osteopontin protein in normal-weight control animals, HFSD-fed animals, and HFSD-fed and EMD-treated animals. Representative immunohistochemistry images are shown. (**b**) Mean intensity of osteopontin in normal-weight control animals, HFSD-fed animals, and HFSD-fed and EMD-treated animals. Bars show mean ± SEM; *n* = 5 animals/group; * significant (*p* < 0.05) difference between groups. (**c**) Frequency distribution of different intensity categories for osteopontin in normal-weight control animals, HFSD-fed animals, and HFSD-fed and EMD-treated animals. The intensity of immunohistochemical staining was assigned to five intensity categories (1 = very low, 2 = low, 3 = moderate, 4 = high, 5 = very high). EMD (enamel matrix derivative), HFSD (high fat, high sucrose diet), P (pulp), D (dentin), PDL (periodontal ligament), B (bone).

Unlike RUNX2, osteopontin was detected most strongly in the HFSD group without EMD treatment. The lowest mean staining intensity was observed for the control group, followed by the HFSD group with EMD application (Figure 4a,b). The mean osteopontin intensity was significantly (*p* < 0.05) different from the control group and the HFSD group with EMD application (Figure 4b). As shown in Figure 4c, in terms of osteopontin intensity, only the three highest categories were found in the HFSD group, whereas the control group was characterized by only the three lowest intensity categories (Figure 4c).

## 3. Discussion

Although the association between periodontitis and obesity is well explored, it is unclear whether obesity is associated with a worse therapeutic outcome after periodontal treatment [32,33]. Our animal study with establishment of fenestration defects in rat mandibles showed that spontaneous healing of periodontal defects was compromised in obesity. The application of regeneration-promoting molecules, i.e., EMD, partially compensated for the negative effect of obesity. Nevertheless, EMD-stimulated bone healing in obese animals was not better than the spontaneous healing in the obesity-free control group, indicating that obesity may also inhibit the stimulatory effects of regeneration-promoting molecules. Overall, these results show that obesity can negatively influence bone healing processes and suggest that the response to periodontal therapy may be compromised in humans (Figure S1; supplementary material).

Obesity is a disease characterized by abnormal or excessive fat accumulation that poses a health risk [34]. Numerous meta-analyses have shown that periodontitis is associated with obesity, i.e., periodontitis patients are more likely to be overweight or obese compared to periodontally healthy individuals [29,30]. Conversely, overweight or obese individuals are more likely to have periodontal disease compared to normal weight subjects [29,30]. Although more research is needed to better understand the nature of this association, it can be assumed that it is a bidirectional relationship, meaning that obesity may contribute to the initiation and progression of periodontitis, but periodontitis may also promote the development of obesity [31]. In addition, there are also common risk factors that may contribute to both periodontitis and obesity [31].

Among the molecules that can promote periodontal regeneration are the enamel matrix proteins [17]. These can be extracted from porcine tooth germs and commercially purchased as enamel matrix derivative (EMD) for clinical use [18]. It has been clearly demonstrated on human biopsies that EMD promotes periodontal regeneration [19,20]. For a long time, it was unclear how the regeneration-promoting effects of EMD were achieved. However, a number of preclinical studies have deciphered the mechanisms and demonstrated that EMD promotes proliferation and migration of periodontal cells and stimulates the synthesis of growth and differentiation factors as well as mineralization [17,21]. Furthermore, the antibacterial effects of EMD or the vehicle material could also be partly responsible for the regeneration-promoting effects [22,23]. Nevertheless, some preclinical studies, in particular, have also revealed that the regeneration-promoting effects of EMD may be diminished in the presence of inflammation, bacterial infection, and tobacco smoke/nicotine [24,25,26].

Obesity seems to be associated with compromised wound healing after surgery [35,36]. A recent review shows that a high-fat diet has an impact on bone formation and resorption. According to this review, obese mice exhibit increased bone resorption associated with increased serum TRAP levels in osteoblasts and decreased *RUNX2/Cbfa1* levels [37]. These data are in accordance with our results, showing a higher number of TRAP+ cells in the HFSD group and lower mean intensity of RUNX2. The potential of EMD to promote osteogenesis and synthesis of RUNX2 offers a possible explanation for the higher intensity of RUNX2 in the HFSD group with EMD treatment as compared to HFSD animals without EMD [38]. Osteopontin is produced by osteoblasts and osteoclast, thus involved in bone formation and remodeling. Osteopontin is also overexpressed in adipose tissue of obese individuals [39]. Thus, osteopontin also appears to play a pro-inflammatory role, which may provide a possible explanation for the higher intensity of osteopontin in the bone of the HFSD group when compared to the other two groups. Further studies are needed to elucidate the exact impact of osteopontin on bone regeneration.

Since plasma levels of certain adipokines are altered in obesity, we have extensively investigated their influence on periodontal cells in previous studies. Adiponectin, which is reduced in plasma in obesity, significantly stimulated growth factor and extracellular matrix expression, proliferation, and in vitro wound healing, significantly reduced constitutive tumor necrosis factor-α expression and caused significant upregulation of its own expression [40,41]. The reduced adipokine levels in obesity could therefore contribute to decreased periodontal matrix and bone formation. Furthermore, we also demonstrated that the beneficial effects of EMD on a number of cellular functions critical for periodontal regeneration are partially enhanced by adiponectin [41]. An obesity-induced decrease in adiponectin could therefore impair EMD-stimulated periodontal regeneration, which is very much in agreement with this animal study, in which the use of EMD in obesity did not result in better healing than in the control group. Elevated levels of pro-inflammatory adipokines such as leptin, visfatin and resistin, as found in obese individuals, may represent another crucial pathomechanistic link for how obesity might contribute to compromised periodontal healing [31,42]. In an in vitro study by our group, leptin caused significant downregulation of growth and transcription factors and matrix molecules and inhibited SMAD signaling under regenerative conditions induced by EMD [43]. Obese individuals also exhibit increased circulating levels of visfatin [44]. EMD-stimulated PDL cells were therefore also incubated with visfatin in previous experiments [45]. EMD stimulated the expression of growth factors and their receptors, matrix molecules and osteogenesis-associated factors and promoted wound closure and calcium accumulation. However, in the presence of visfatin, all these stimulatory effects were diminished [45]. Therefore, these in vitro results also suggested that obesity impairs the regenerative capacity of PDL cells and, thus, periodontal healing, which is in agreement with this in vivo study. Resistin also appears to affect the metabolism of periodontal soft and hard tissues by reducing markers of matrix formation and bone tissue [46].

Moreover, the hormone somatostatin (SST) exerts antiproliferative, antiangiogenic and proapoptotic effects by binding to its receptors, such as SSTR2. We have been able to show that leptin and visfatin lead to upregulation of *SSTR2* in periodontal cells, and via this pathway periodontal healing may be diminished [47]. Tyrosine hydroxylase (TH) catalyzes the rate-limiting step in the synthesis of catecholamines and is associated with increased periodontal disease progression under chronic stress. In our in vitro experiments, *TH* gene expression and TH protein levels were increased by leptin and visfatin [48]. These results also suggest that this may represent a pathomechanism by which obesity exerts a negative influence on periodontal homeostasis. Taken together, adipokines could be a pathomechanistic link whereby obesity and obesity-related diseases enhance the risk for periodontitis and compromised periodontal healing [38].

Obesity is characterized by chronic systemic inflammation. Interestingly, previous studies have been able to show that the effects of EMD are reduced under inflammatory conditions, which again is in line with the results from this animal study [24,49].

Interestingly, EMD was able to partially compensate for the negative effects of obesity on osseous healing, osteoclast number, RUNX2 and osteopontin in this study. This may have been in part because EMD exhibits anti-inflammatory effects that could have reduced the pro-inflammatory processes induced by obesity in this study [50].

In this animal study, Wistar rats were used, in which obesity was induced by HFSD. Our previous analyses have demonstrated that this obesity model is suitable for studying the effect of obesity on periodontal tissues, although this model has some limitations [51]. Further clinical studies in patients will show whether our results from animal experiments can be extrapolated to the human situation.

In our study, a widely used fenestration defect model was applied to analyze bone healing [52]. The fenestration model is mainly suitable for the study of regenerative processes and is a very well-established model for such studies. Since we wanted to study the effects of regeneration-promoting enamel matrix proteins, we decided to use this model. Periodontal regeneration, as a form of periodontal healing, involves not only the restoration of the form, structure and function of the hard tissues, but also of the soft tissues such as the periodontal ligament. In the present study, however, the focus was on bone healing. Future studies should also address the regeneration of soft tissue structures. The model we chose here did not involve periodontal pockets that occur in naturally occurring or experimentally induced periodontitis. Therefore, the conclusions from our study must be drawn with caution. Future studies should show whether the unfavorable effect of obesity on bone healing observed here is also detectable in the treatment of periodontal pockets. In our fenestration model, bone healing occurred in a closed situation, unlike periodontal pocket therapy, i.e., the wound was sutured after the surgical procedure so that the bone defect was largely protected from bacterial infection. However, our previous experiments have been able to show that obesity can negatively influence periodontal homeostasis even in the absence of clinically visible periodontal infection [53]. The fenestration defect model used in our study was a defect that allowed spontaneous healing. On the one hand, this was a disadvantage because it made the regenerative effect of EMD less obvious. On the other hand, however, this also allowed the investigation of obesity on spontaneous defect healing.

A recent study has demonstrated that soft tissue healing is delayed in prediabetic mice fed a high-fat diet [54]. This observation is consistent with our data, which also showed impaired wound healing. Another in vivo study revealed that osseointegration of dental implants is impaired in animals suffering from obesity/metabolic syndrome or uncontrolled diabetes [55]. These findings again concur with our results and confirm that obesity leads to delayed or impaired bone healing. Further preclinical studies are needed to elucidate the pathomechanisms underlying this phenomenon.

Another limitation of our study was that all animals were sacrificed after 4 weeks. Thus, it could not be clarified whether obesity only delayed bone healing or permanently inhibited it. Additional time points of examining bone healing, number of TRAP+ cells, RUNX2 and osteopontin production would therefore have been interesting but would also have required more animals. Additional studies should also examine the influence of obesity on other matrix molecules, inflammatory mediators and cells and signaling cascades in bone healing.

**Figure 5:**
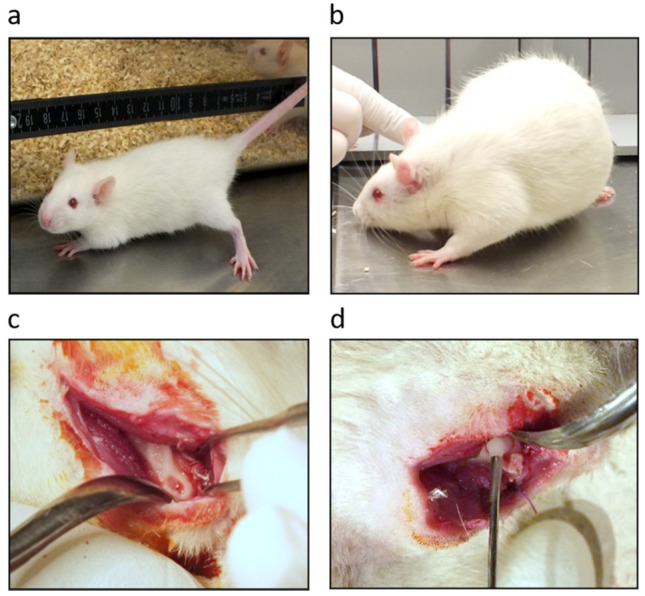
Obesity and bone fenestration-type defect model. (**a**) Wistar rat at baseline and (**b**) after high-fat, high sucrose diet (HFSD). Fenestration-type defect of alveolar bone over the root of a mandibular first molar (**c**) and application of enamel matrix derivative (EMD) (**d**).

In our study, a standardized fenestration defect was created with an approximate height and depth of 2 mm and a horizontal dimension of 4 mm (Figure 5c). To ensure standardization of the defects, they were checked during surgery with a periodontal probe (UNC 15, Hu-Friedy) and corrected if necessary. Nevertheless, it cannot be completely ruled out that the defects varied minimally.

Our study shows some technical limitations. Blocks were manually embedded, and the transversal plane was controlled in hematoxylin and eosin (H&E) stained slides using light microscopy. In the case of an incorrect plane, blocks were melted and embedded with a new orientation until the right transversal plain was achieved.

In our study, EMD was used to stimulate regenerative healing. EMD contains enamel matrix proteins, of which amelogenin is the most frequent [56]. However, numerous studies have also shown that in addition to these enamel matrix proteins, EMD contains growth and differentiation factors, e.g., transforming growth factor-β1, which also contribute to the regenerative effects of EMD [57]. Thus, it is also not surprising that the stimulatory effects of EMD are mediated via both the MAPK and SMAD signaling cascades. Future studies could also investigate the effect of obesity on periodontal regenerative healing promoted by membranes, bone grafts and/or bone substitute materials.

## 4. Materials and Methods

### 4.1. Animal Model

Four-week-old Wistar rats were obtained from Charles River Laboratories (Sulzfeld, Germany) and maintained in the University of Bonn animal facility according to institutionally approved protocols. Approval for the animal study was obtained from the University of Bonn and local authorities (Landesamt für Natur, Umwelt und Verbraucherschutz Nordrhein-Westfalen; Az 87-51.04.2010.A394). All applicable international, national and/or institutional guidelines for the care and use of animals were followed. A total of 15 animals (*n* = 5/group) were housed under stable environmental conditions at a room temperature of 21 °C and humidity of 35% on a 12-h day-night cycle. All animals received water and food ad libitum. The animals were divided into the following experimental groups: (1) control animals, (2) animals fed HFSD (sniff, Soest, Germany) and (3) animals whose defects were treated with EMD (Emdogain^®^, Straumann, Basel, Switzerland) and fed HFSD.

### 4.2. Induction of Obesity

To induce obesity, 10 animals received a normal diet until week 4, followed by an HFSD characterized by high fat content (25.1% fat) and high sucrose content (28.8% sucrose) (sniff) until the end of the study [51] (Figure 5a,b). Animals in the control group received a standard diet (sniff) throughout the experimental period. The body weight of all animals was recorded weekly. After 4–5 weeks of HFSD diet, the animals of the HFSD-only group and the combined HFSD/EMD group reached a mean body weight of 476.2 ± 17.64 g and 453.6 ± 14.99 g, respectively, whereas the control group had a mean weight of 418.72 ± 16.3 g. At this time point, bony fenestration defects were surgically created in all animals. All animals were sacrificed 4 weeks after surgery. At this time point, the mean weight of animals in the HFSD-only group was 554.80 ± 30.36 g and that of animals in the combined HFSD/EMD group was 527.80 ± 14.24 g. The animals of the control group weighed 499.58 ± 9.60 g.

### 4.3. Periodontal Fenestration-Type Defect Model and Treatment with EMD

A fenestration-type defect was created to investigate the effects of obesity on regenerative bone healing [52]. Animals were anesthetized intraperitoneally with ketamine 10% (75 mg/kg) and medetomidine (0.5 mg/kg). Local anesthesia was performed with articaine 1:200,000 dissolved in saline according to the weight of the animal. The skin on the right side of the jaw was shaved and disinfected with liquid povidone iodine. Using a 15-c scalpel, a 2-cm skin incision was made at the inferior border of the mandible. After cutting the superficial fascia, the underlying masseter muscle and periosteum were separated from the bone to expose the right mandible. After careful dissection with a raspatory, the oral mucosa was identified on the upper wall of the surgically created access chamber without violating the attachment of the intraoral keratinized gingival margin. Bone removal on the buccal aspect of the first molar was performed using a slow-speed rose bur under saline irrigation. A standardized fenestration defect was created with an approximate height and depth of 2 mm and a horizontal dimension of 4 mm (Figure 5c). In order to standardize the dimensions of the defect, this was measured during surgery using a standardized periodontal probe (UNC 15, Hu-Friedy, Frankfurt am Main, Germany). The distance between the upper margin of the bony defect and the crestal bone of the first molar was 1 mm. In addition, the buccal root of the first molar was freed from its periodontal ligament, cementum and superficial dentin. Root debridement was performed with saline followed by conditioning with ethylenediaminetetraacetic acid (EDTA, PrefGel^®^, Straumann) for two minutes according to the manufacturer’s instructions. After two minutes, the wound was thoroughly rinsed with saline. In half of the animals fed with HFSD, fenestration defects were treated with enamel matrix derivative (EMD, Emdogain^®^, Straumann) (Figure 5d). EMD was applied with a sterile syringe provided by the manufacturer so that the complete defect was covered.

Wounds were closed with absorbable suture material (4.0 Vicryl, Ethicon, Johnson & Johnson Medical, Norderstedt, Germany) in two layers, one layer on the muscles and one on the skin. All animals received carprofen (5 mg/kg) as an analgesic. Wound healing, swelling and weight of the animals were monitored every other day for one week after surgery. No complications, such as wound infections, wound healing abnormalities, unnatural weight loss or death, occurred after surgery in any of the animals. All animals were sacrificed 4 weeks after surgery. The skulls of the rats were removed and stored in 4% phosphate-buffered formaldehyde (Merck, Darmstadt, Germany) for fixation.

### 4.4. Histological Preparation and Staining

After fixation, the skulls were decalcified in 10% EDTA solution (EMD Millipore, Billerai, MA, USA) for 12 weeks. Subsequently, the right mandible was separated from the rest of the skull and processed for paraffin histology. After dehydration in an ascending ethanol series, the tissue was embedded in paraffin. Sections with a thickness of 2.5 µm were cut and mounted on glass slides (Engelbrecht, Edermünde, Germany) and dried overnight at 37 °C. The sections were cut in a transverse plane across the entire coronal to apical width of the defect to provide a buccal-lingual view of the defect. Before reaching the region of interest, sections were stained with hematoxylin and eosin (H&E; Merck) and controlled using light microscopy in order to recognize the correctness of the transverse plane. In case the plane was not correct, blocks were melted and embedded until the correct plane was reached. Once the sections reached the area of the defect, all slides were collected and stored for further examination. Every tenth section was stained with H&E. After microscopic identification of the coronal, middle and apical levels of the defect, three sections corresponding to the three levels were selected for further examination (*n* = 3/animal and *n* = 15/group, respectively). For osteoclast identification, three serial sections corresponding to those used for H&E staining were selected and stained with TRAP (Sigma-Aldrich, Taufkirchen, Germany) according to the manufacturer’s instructions (Figure S2; Supplementary Material).

### 4.5. Immunohistochemistry

Detection of RUNX2 and osteopontin was performed by immunohistochemistry. For both antibodies, three serial sections from each animal were deparaffinized and rehydrated, followed by blocking endogenous peroxidase with 0.3% methanol (Merck)/30% H_2_O_2_ (Merck) in the dark for 10 min. Sections for RUNX2 detection were pretreated with pepsin at 37 °C for 20 min, followed by pre-blocking with 1× tris-buffered saline (Merck)/4% bovine serum albumin (Merck) at room temperature for 1 h. In the next step, the sections were incubated with a rabbit polyclonal antibody against RUNX2 (ab23981, abcam, Cambridge, UK) and a rabbit polyclonal anti-osteopontin antibody (ab8448, abcam). Incubation for RUNX2 was performed in a humid chamber at 4 °C overnight and for osteopontin at room temperature for 1 h. Sections were then rinsed and incubated for 30 min at room temperature with a goat anti-rabbit IgG-HRP secondary antibody (Dako, Hamburg, Germany). 3,3-diaminobenzidine chromogen (Thermo Fisher Scientific, Dreieich, Germany) was used to visualize peroxidase activity. Sections were then rinsed and counterstained for 30 s with Mayer’s hematoxylin (Merck) (Figure S2; Supplementary Material).

### 4.6. Histomorphometric Analysis

Images of all sections were obtained using the Axioskop 2 microscope and AxioVision 4.7 software (Carl Zeiss, Jena, Germany) at 10× magnification. The region of interest (ROI) was selected by including the root of the first molar and the surgical defect. In order to standardize measures and minimize variations within measurements, a quadrant with standardized dimensions, including root, PDL and bone of the buccal site of the first molars, was positioned on each image. The ROI was cut out within the limits of the quadrant and used for further evaluations. Images of H&E with the standardized quadrants were used as a template to ensure that the quadrant was placed in the same position for all other pictures used for TRAP, RUNX2 and osteopontin staining. In this way, variations were minimized. The open-source programs ImageJ and Fiji Plug-in were used to quantify the measurements [58,59]. Briefly, in H&E-stained slides, bone within the ROI was selected and quantified. Measurements were performed by two independent calibrated scientists. Both scientists performed measurements blinded. For statistical analysis, the percentage ratio of bone area to total area of the ROI was calculated. After TRAP staining, the number of TRAP+ bone cells within the ROI was manually counted, and the values were further used for statistical analysis. After immunohistochemical staining with anti-RUNX2 (ab23981, abcam) or anti-osteopontin (ab8448, abcam) antibody, the intensity of staining within the ROI was recorded using the Fiji plug-in. Values were assigned to five intensity categories (1: very low, 2: low, 3: moderate, 4: high, 5: very high) and subsequently analyzed (Figure S2; Supplementary Material).

### 4.7. Statistical Analysis

Statistics were performed using the IBM SPSS Statistics Software (Version 23, IBM SPSS, Chicago, IL, USA). Mean values and standard errors of the means were calculated and homogeneity of variances as well as normal distribution were examined. Mann-Whitney U and chi-squared tests were applied to reveal statistically significant differences between the groups (*p* < 0.05).

## 5. Conclusions

In summary, our animal study showed that obesity inhibited bone healing. Application of EMD partially compensated for the negative effect of obesity. Nevertheless, the EMD-stimulated bone healing in obesity was not better than the spontaneous healing in the obesity-free control group, indicating that obesity may also inhibit the stimulatory effect of regeneration-promoting substances. Our results suggest that the response to periodontal therapy may be compromised in obese patients compared to normal-weight individuals.

## Data Availability

Data sharing is not applicable to this article.

## Supplementary Materials

The following are available online at https://www.mdpi.com/article/10.3390/ijms222413339/s1.

## Abbreviations

B	bone
D	dentin
EDTA	ethylenediaminetetraacetic acid
EMD	enamel matrix derivative
H& E	hematoxylin and eosin
HFSD	high-fat, high-sucrose diet
MAPK	mitogen-activated protein kinases
P	pulp
PDL	periodontal ligament
ROI	region of interest
RUNX2	runt-related transcription factor 2
SMAD	acronym from small and mothers against decapentaplegic
SST	somatostatin
SSTR2	somatostatin receptor 2
TH	tyrosine hydroxylase
TRAP	tartrate-resistant acid phosphate
