# Supplementary material for: Effects of Obesity on Bone Healing in Rats
Source: Int J Mol Sci. 2021 Dec 11;22(24):13339. doi: 10.3390/ijms222413339 (PMC8704371; doi:10.3390/ijms222413339)
Supplement: Supplementary file 1 [file ijms-22-13339-s001.zip › ijms-1465654-supplementary.pdf]

International Journal of *Molecular Sciences*

MDPI

Article

Effects of Obesity on Bone Healing in Rats

Anna Damanaki [1,*], Svenja Memmert [2], Marjan Nokhbehsaim [3], Ali Abedi[1], Birgit Rath-Deschner [2], Andressa Nogueira [1] and James Deschner [1]

[1] Department of Periodontology and Operative Dentistry, University Medical Center, University of Mainz, 55131 Mainz, Germany; aa_abedi@yahoo.com (A.A.); a.nogueira@uni-mainz.de (A.N.); james.deschner@uni-mainz.de (J.D.)
[2] Center of Dento-Maxillo-Facial Medicine, Department of Orthodontics, University of Bonn, 53111 Bonn, Germany; svenja.memmert@ukb.uni-bonn.de (S.M.); brathde@uni-bonn.de (B.R.-D.)
[3] Section of Experimental Dento-Maxillo-Facial Medicine, Center of Dento-Maxillo-Facial Medicine, University of Bonn, 53111 Bonn, Germany; m.saim@uni-bonn.de (M.N.)
* Correspondence: adamanak@uni-mainz.de; Tel.: +49 (0) 6131-17-5419

Abstract: Although the association between periodontitis and obesity is well explored, it is unclear whether obesity is associated with a worse therapeutic outcome after periodontal treatment. The aim of this study was to investigate the effects of obesity on bone healing with and without the application of regeneration-promoting molecules. A standardized bone fenestration-type defect was created over the root of the mandibular first molar in 15 Wistar rats. Ten animals received a high-fat, high-sucrose diet (HFSD), while the remaining five animals were fed a standard diet. During surgery, the fenestration defects from half of the HFSD-fed, i.e., obese animals, were treated with regeneration-promoting molecules (enamel matrix derivative; EMD). After four weeks, bone healing was evaluated by histomorphometry, TRAP staining and immunohistochemistry for RUNX2 and osteopontin. The analyses revealed that the spontaneous healing of the periodontal defects was compromised by obesity. Application of EMD partially compensated for the negative effect of obesity. Nevertheless, EMD-stimulated bone healing in obese animals was not better than the spontaneous healing in the obesity-free control group, indicating that obesity may also inhibit the stimulatory effects of regeneration-promoting molecules. Our results show that obesity can negatively influence bone healing and suggest that bone healing may be compromised in humans.

Keywords: periodontal regeneration; obesity; fenestration; animal; periodontitis

Citation: Damanaki, A.; Memmert, S.; Nokhbehsaim, M.; Abedi, A.; Rath-Deschner, B.; Nogueira, A.; Deschner, J. Effects of Obesity on Bone Healing in Rats. *Int. J. Mol. Sci.* **2021**, *22*, 13339. https://doi.org/10.3390/ijms222413339

Academic Editor: Christopher W Cutler

Received: 31 October 2021
Accepted: 7 December 2021
Published: 11 December 2021

Publisher's Note: MDPI stays neutral with regard to jurisdictional claims in published maps and institutional affiliations.

Copyright: © 2021 by the authors. Licensee MDPI, Basel, Switzerland. This article is an open access article distributed under the terms and conditions of the Creative Commons Attribution (CC BY) license (https://creativecommons.org/licenses/by/4.0/).

Supplementary material

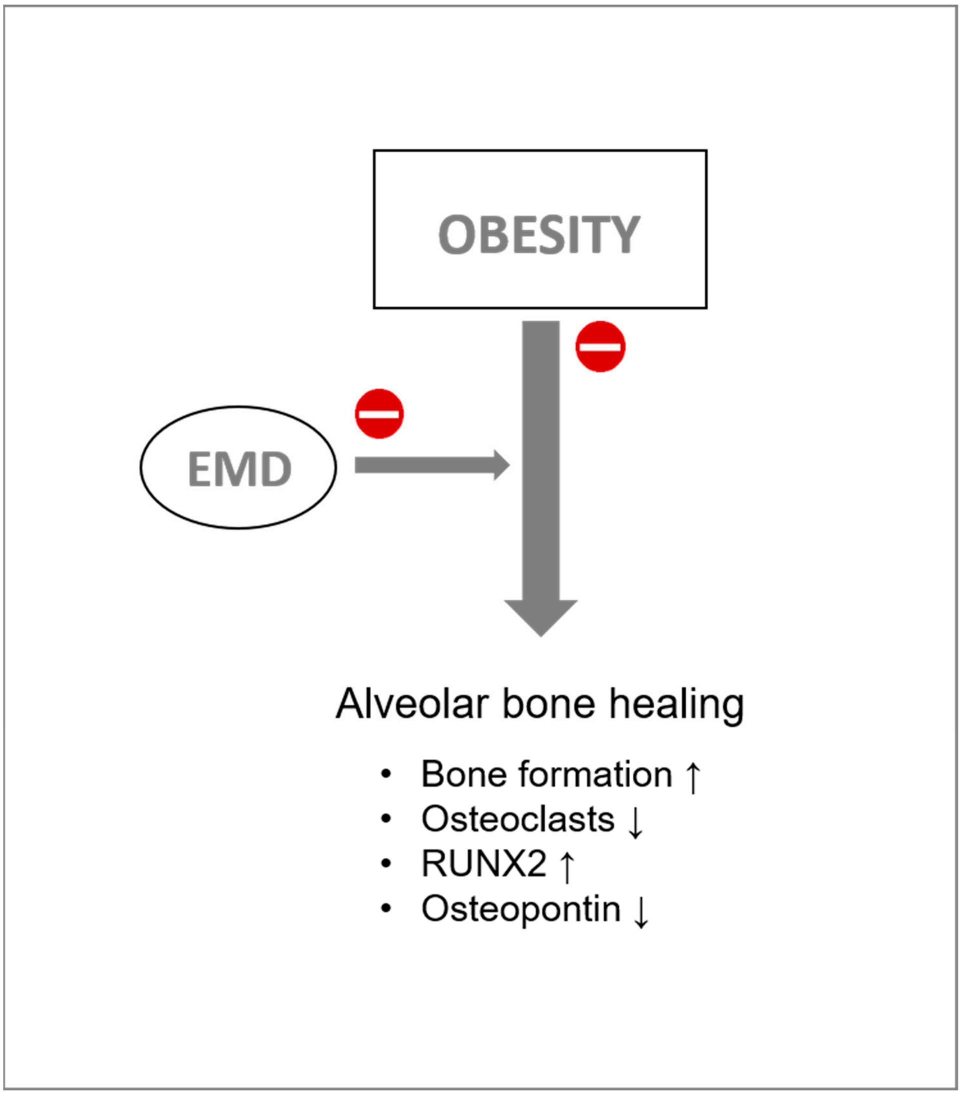

Figure S1. Scheme depicting the impact of obesity and/or EMD on alveolar bone healing in the present study.

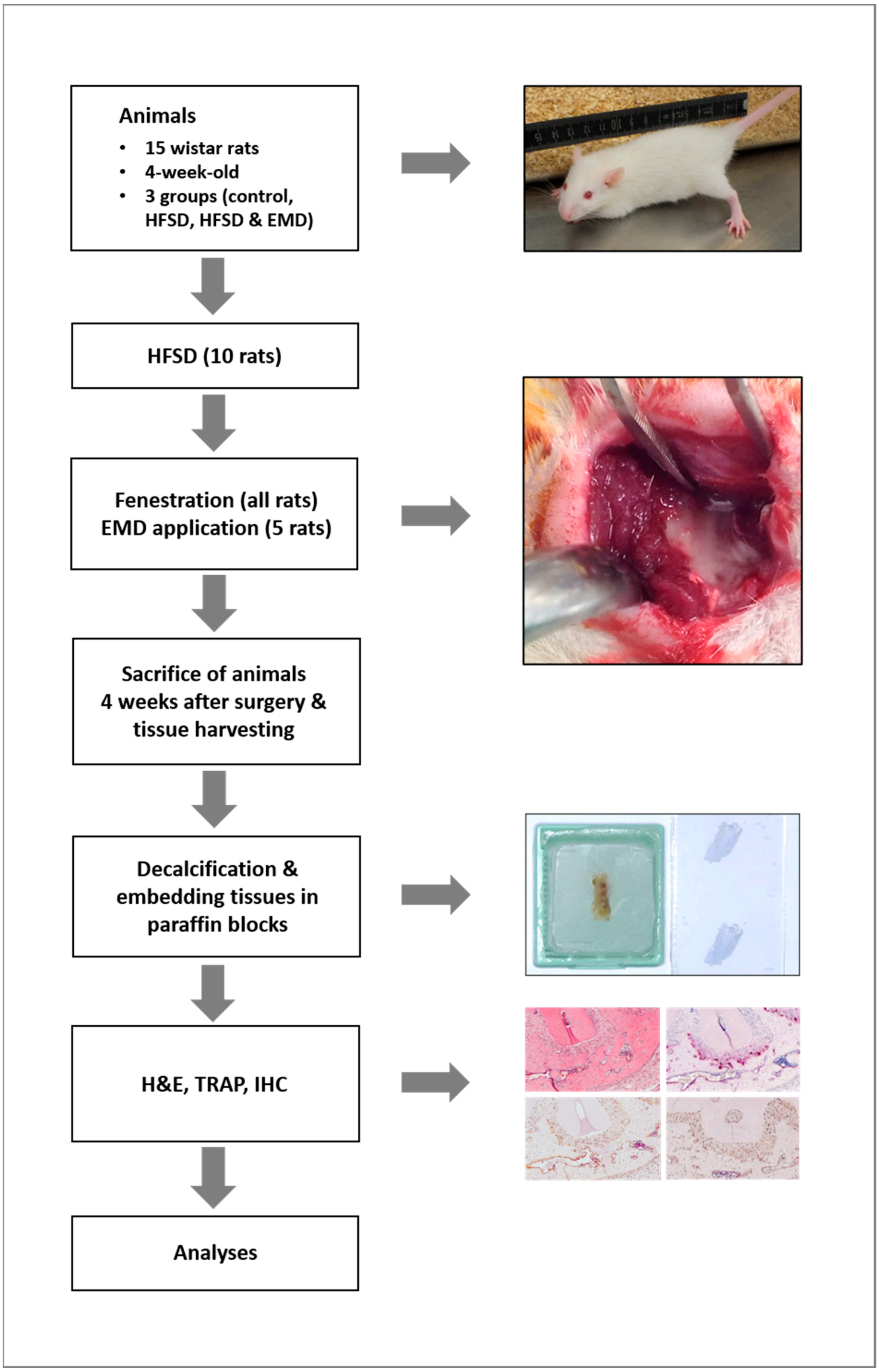

Figure S2. Scheme of the experimental protocol.
